# Estimation of the Radon Risk Under Different European Climates and Soil Textures

**DOI:** 10.3389/fpubh.2022.794557

**Published:** 2022-02-17

**Authors:** Sara Gil-Oncina, Javier Valdes-Abellan, Concepcion Pla, David Benavente

**Affiliations:** ^1^Department of Earth and Environmental Sciences, University of Alicante, Alicante, Spain; ^2^Department of Civil Engineering, University of Alicante, Alicante, Spain

**Keywords:** radon risk, Köppen climate classification, soil hydraulic properties, geogenic radon potential, radon soil transport

## Abstract

Radon is a radioactive gas produced from the natural radioactive decay of uranium and is found in almost all rocks and soils. In confined places (e.g., dwellings, workplaces, caves, and underground mines), radon may accumulate and become a substantial health risk since it is considered the second most important cause of lung cancer in many developed countries. Radon risk assessment commonly considers either field or estimate values of the radon concentration and the gas permeability of soils. However, radon risk assessment from single measurement surveys to radon potential largescale mapping is strongly sensitive to the soil texture variability and climate changes, and particularly, to the soil water content dynamic and its effect on soil gas permeability. In this paper, the gas permeability of soils, and thus, the estimation of radon risk, is studied considering the effect of three different climates following the Köppen classification and four soil textures on soil water content dynamics. This investigation considers the CLIGEN weather simulator to elaborate 100-year length climatic series; Rosseta 3 pedotransfer function to calculate soil hydraulics parameters, and the HYDRUS-1D software to model the dynamics of water content in the soil. Results reveal that climate strongly affects gas permeability of soils and they must be considered as an additional factor during the evaluation of radon exposure risk. The impact of climate and texture defines the soil water content dynamic. Coarse soils show smaller gas permeability variations and then radon risk, in this case, is less affected by the climate type. However, in clay soils, the effect of climate and the differences in soil water content derive in gas permeability variations between 100 and 1,000 times through an annual cycle. As a result, it may cross the boundary between two radon risk categories. Results deeply confirm that both climate and texture should be compulsory considered when calculating the radon exposure risk and in the definition of new strategies for the elaboration of more reliable geogenic radon potential largescale maps.

## Introduction

Radon, ^222^Rn or just Rn, is a radioactive gas naturally present in almost all rocks and soils of the Earth's surface and it is the most important source of ionising radiation among those that are of natural origin (1). Radon comes from the Radium disintegration, ^226^Ra, which is a member of the disintegration from the ^238^U chain, and its half-life is 3.825 days. Radon has a variety of geoscientific applications, ranging from its utilisation as a potential earthquake precursor and proxy of tectonic stress, specifically in volcanic environments, to a wide range of applications as a tracer in marine and hydrological settings and global warming investigations (2).

In confined places (e.g., dwellings, workplaces, caves, and underground mines), however, radon may accumulate to higher concentrations where it may pose a substantial health risk (3). Thus, the World Health Organisation (1) stated that Rn is the second most important cause of lung cancer after smoking in many developed countries. Particularly, the exposure to the alpha particles from the radon and its short-lived progeny decays does not only affect the lung tissues, but also the skin outer layer where is irradiated as the first barrier in the human body (4–6).

The origin of most indoor radon however is the geology under and around buildings, where uranium-bearing soils and rocks provide a source of radon gas, and where transport to the surface may be facilitated by permeable superficial deposits (7). Radon risk assessment can be evaluated at different scales and varies from direct measurements of indoor radon, soil gas measurements, chemical analysis of bedrock and soils, and airborne eU (equivalent uranium) measurements (7). Geogenic radon potential, GRP, considers that the major source of indoor radon concentration is the soil gas radon (8). One of the most recognised methods for the GRP calculation of an area was established by Neznal (9). It considers field measurements of the radon concentration in soil gas and the gas permeability of soils. Thus, high GRP indicates a high probability of radon entering indoors due to geogenic reasons. Radon risk assessment using this method is determined by a single measurement from one season, although the risk for human health due to radon arises from long-term exposure to certain doses. The elaboration of GRP largescale mapping considers also indirect parameters such as radium or uranium content, for radon soil concentration, and the tabulated permeability values depending on the type of lithology. Nevertheless, this methodology does not consider the soil water content and its effect on soil gas permeability, which can be a key factor in the movement of gas through porous media as was clearly stated by Benavente (10). For a specific region, soil water content is closely related to soil texture and climate features, such as precipitation and temperature regimes, which must be considered when dealing with radon mobility in soils and, consequently, in radon risk. Even more, the soil water content dynamics along the different seasons should be considered in the evaluation of the GRP since the soil humidity, and hence the air soil permeability, may change significantly between different seasons.

The main objective of this investigation is to investigate gas permeability and radon risk in four different soil types under three contrasting climates in Europe. Firstly, we analyze the impact of the type of climate and texture on the soil water content dynamic in a soil profile of 2 m deep. Secondly, we determine the gas permeability variation according to the soil water content for all soil-climate combinations. Finally, we discuss the gas permeability variation in terms of radon risk categories to provide new insights in the elaboration of more reliable GRP largescale maps considering the climate and soil texture.

## Materials and Methods

### Weather Time Series

The present study considers three climatic scenarios, according to the Köppen classification trying to represent most common climates in Europe: Bsk, cold semi-arid climate, typical from many regions in South Europe; Csa, temperate Mediterranean climate with dry hot summers and moderate winters, also common in South Europe; and finally, Dfb, humid continental climate with cool winters and moderate summers, typical from central Europe. The Köppen climate system, which has been upgraded over time, classified Planet Earth into five defined zones and 30 sub-types dependent mainly on vegetation criteria and named with two or three-letter words in alphabetical sequence *per se*. The first letter refers to the zone (i.e., B: arid or dry zone, C: warm or mild temperate zone, and D: continental zone), and the second and/or third letters allude to the dryness or temperature (i.e., zone B subdivides into four categories related to regions, i.e., BWh, hot desert climate; BWk, cold desert climate; Bsh, hot semi-arid climate; and Bsk, cold semi-arid climate). The climate classification followed in the present study is described in (11), and the most important differences with the original classification proposed by Köppen (12) can be found in (13).

CLIGEN weather simulator [CLImate GENerator (14)] was used to generate a 100-year length temporal series of daily rainfall, maximum and minimum temperatures, and solar radiation for the selected climates. The input files required by CLIGEN for each climate were obtained from the longest US weather station among all the stations belonging to each specific climate. The complete database of stations input parameters from the United States Department of Agriculture (USDA) can be found on its webpage (https://www.ars.usda.gov/midwest-area/west-lafayette-in/national-soil-erosion-research/docs/wepp/cligen/).

Potential evapotranspiration, ET_0_, was daily calculated with a modified version of the Hargreaves equation (15, 16) using the output results provided by CLIGEN.

**Figure 1:**
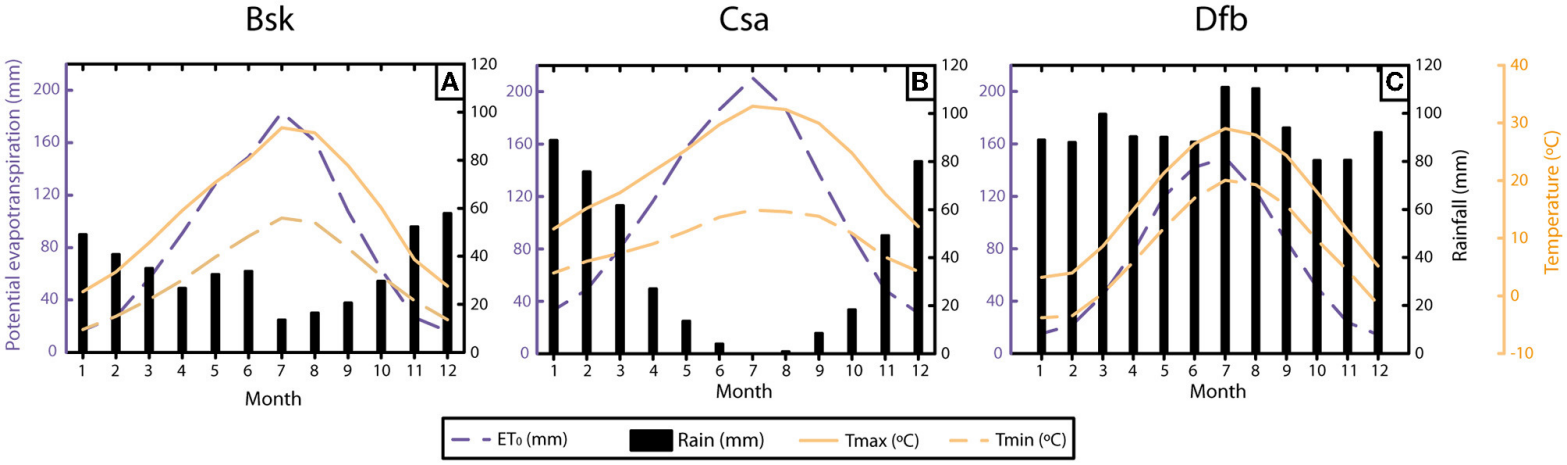
Mean total monthly rainfall, mean monthly maximum and minimum temperature, and mean monthly total potential evapotranspiration for Bsk climate **(A)** Csa climate **(B)** and Dfb climate **(C)**.

Among the 100-year data, the 1st year was used as a warm-up period in order to achieve a stationary soil water profile representative of each climate. This option reduces the importance of the subjective initial soil water content profile at the beginning of the simulation. Figure 1 summarises the main characteristics for each selected climate.

### Soil Texture and Hydraulic Properties

Four different soils were considered according to the USDA texture soil classification to test the water dynamics under different climates: clay soil, silty loam soil, loam soil, and loamy sand soil.

Based on their USDA texture classifications, soil hydraulic parameters were obtained using the Rosseta 3 pedotransfer function, PTF, (17). Rosetta 3 is a new version of the initial PTF developed by Schaap (18), with some improvements such as a covariance matrix between soil water retention properties and saturated water permeability and introducing a new set of hierarchical functions. Soil hydraulic properties can be estimated from standard soil properties with several degrees of complexity.

Rosetta provides soil hydraulic properties of the van Genuchten-Mualem model: the saturated water content, θ*s* [L^3^·L^−3^]; the residual water content θ*r* [L^3^·L^3^]; the saturated hydraulic conductivity Ks [L·T^−1^]; and empirical coefficients that determine the shape of the hydraulic functions, α [L^−1^] and *n* [-] (Table 1). The empirical α and *n* coefficients are crucial to shaping soil hydraulic functions. α is related to air entry value, and *n* to pore size distribution: for 100% sand, sandy soils have an *n* = 4.47; for 100% silt, silt soils have an *n* = 1.67; and clayey soils have an *n* = 1.18 for 100% clay.

**Table 1:** Soil hydraulic parameters obtained from Rosetta 3 for silty loam, loam, clay, and loamy sand soils.

**Soil texture**	**θ_r_ cm^3^·cm^−3^**	**θ_s_ cm^3^·cm^−3^**	**α (cm^−1^)**	***n* (–)**	***K*_s_ (cm/s)**
Silty loam	0.0645	0.4387	0.0051	1.6626	18.26
Loam	0.0609	0.3991	0.0111	1.4737	12.04
Clay	0.0982	0.4588	0.0150	1.2529	14.75
Loamy sand	0.0485	0.3904	0.0347	1.7466	105.12

### Soil Water Content Modelling

Water content, θ, and flow were simulated using the software HYDRUS-1D (19) numerical solution. This code solves the Richards equation (20) for the saturated water flow variable according to:


(1) $$\begin{array}{l} \frac{\partial \theta }{\partial t}=\frac{\partial }{\partial z}\left(K\left(h\right)\left(\frac{\partial h}{\partial z}+1\right)\right)-S\left(h\right) \end{array}$$

where *t* is time [T]; *z* is the vertical coordinate [L]; *K*(*h*) is the soil unsaturated hydraulic conductivity [L·T^−1^]; and *S*(*h*) is the sink term that represents water uptake by plants [L^3^·L^−3^·T^−1^].

The soil unsaturated hydraulic conductivity was defined with the van Genuchten-Mualem constitutive relationships (21, 22).

A 200 cm-deep vertical soil domain was considered for all simulations and soil-climate combinations. The vertical discretization of the profile started with two 0.1-cm numerical elements, two 0.2-cm elements, two 0.3-cm elements, and so on until obtaining a 1-cm length. The distance of 1 cm length between numerical nodes was kept to the end of the profile. Variable atmospheric conditions were set at the top boundary using the weather time series results, and free drainage was imposed on the bottom boundary as implemented in HYDRUS-1D. Observation nodes were defined at 10:10:100 depths to obtain temporal series of soil water content.

### Gas Permeability in Unsaturated Soils

Radon permeability decreases as water content increases, which varies the potential risk of radon exposure. The presence of water in the porous materials reduces both connected porosity and pore size and increases the tortuosity (23). The calculation of unsaturated gas permeability is based on the methodology proposed by Benavente (10), as follows:

a) Calculation of gas permeability: we transform hydraulic conductivity (depends on both porous media and fluid properties) to water permeability (depends on porous media), i.e., $k_{w}\left(m^{2}\right)=1.1810^{-14}K_{w}(cm\cdot day^{-1})$ pure water at 20°C (Table 1). Then we assume Darcy's law that considers gas and liquid water permeabilities to be equal (*k*_*g*_ = *k*_*w*_).
b) Influence of water content: the unsaturated gas permeability is calculated as a function of the effective water saturation obtained from HYDRUS-1D for all soil-climate combinations at different soil depths. Using the soil hydraulic properties of the van Genuchten-Mualem model derived from Rosseta 3 pedotransfer function, we obtained the unsaturated gas permeability as follows:

(2) $$\begin{array}{l} k_{g}\;\left(S_{w}\right)=k_{g}\left(1-\;S_{w}^{\tau }\right)\;\left[1-{\left(1-{\left(1-S_{w}^{m^{-1}}\right)}^{m}\right)}^{2}\right] \end{array}$$

where the water saturation, S_w_, is defined as:


(3) $$\begin{array}{l} S_{w}=\frac{\;\theta \left(h\right)\;\theta_{r}}{\theta_{s}-\;\theta_{r}} \end{array}$$

where h is the soil pressure head [L] and θ is the actual volumetric water content [cm^3^·cm^−3^], and α [L^−1^], n [-], and τ [-] are empirical coefficients that determine the shape of the hydraulic functions. The tortuosity value of τ = 0.5 is commonly assumed, based on (15); parameter m [-] was calculated as a function of *n*, *m* = 1 − *n*^−1^.

c) Permeability is eventually expressed as:

(4) $$\begin{array}{l} pk_{g}\;=\;-log\;\left(k_{g}\right) \end{array}$$

where high pk_g_ values mean impermeable soils, whereas low pk_g_ values imply permeable soils. The methodology proposed by Benavente (10) was validated using the database of the Canadian component of the North American Soil Geochemical Landscapes Project (NASGLP), which were addressed to assess the radon potential risk included in Health Canada's National Radon Program (24).

## Results

### Climate and Soil Water Content Values

The temporal series for the cold semi-arid climate (Bsk) reports an average total annual precipitation of 407 mm and potential evapotranspiration, ET_0_, of 1,027 mm showing a clear higher water demand over the total precipitation (Figure 1A). The consequence of this feature is the dominance of dry soils along most months during the year. This situation increases the movement of gas through soils and even favours the development of shrinkage cracks in swelling soils, which will enhance the radon movement.

This fact of higher ET_0_ than precipitation is also observed in the temperate Mediterranean climate (Csa), with more than 1,300 mm of ET_0_ and 427 mm of average total annual precipitation (Figure 1B). The case of the humid continental climate (Dfb) is completely different, with more than 1,100 mm of average total annual precipitation and total annual ET_0_ below 900 mm (Figure 1C). The average maximum annual temperature for the three climates was 14.4, 22.6, and 16.2°C for the climates Bsk, Csa, and Dfb, respectively. Focusing on the temporal distribution of the precipitation, Csa climate shows the most uneven behaviour with almost no precipitation during the summer months and 60% of the precipitation concentrated in just 3 months, which is a particular feature of the region.

**Figure 2:**
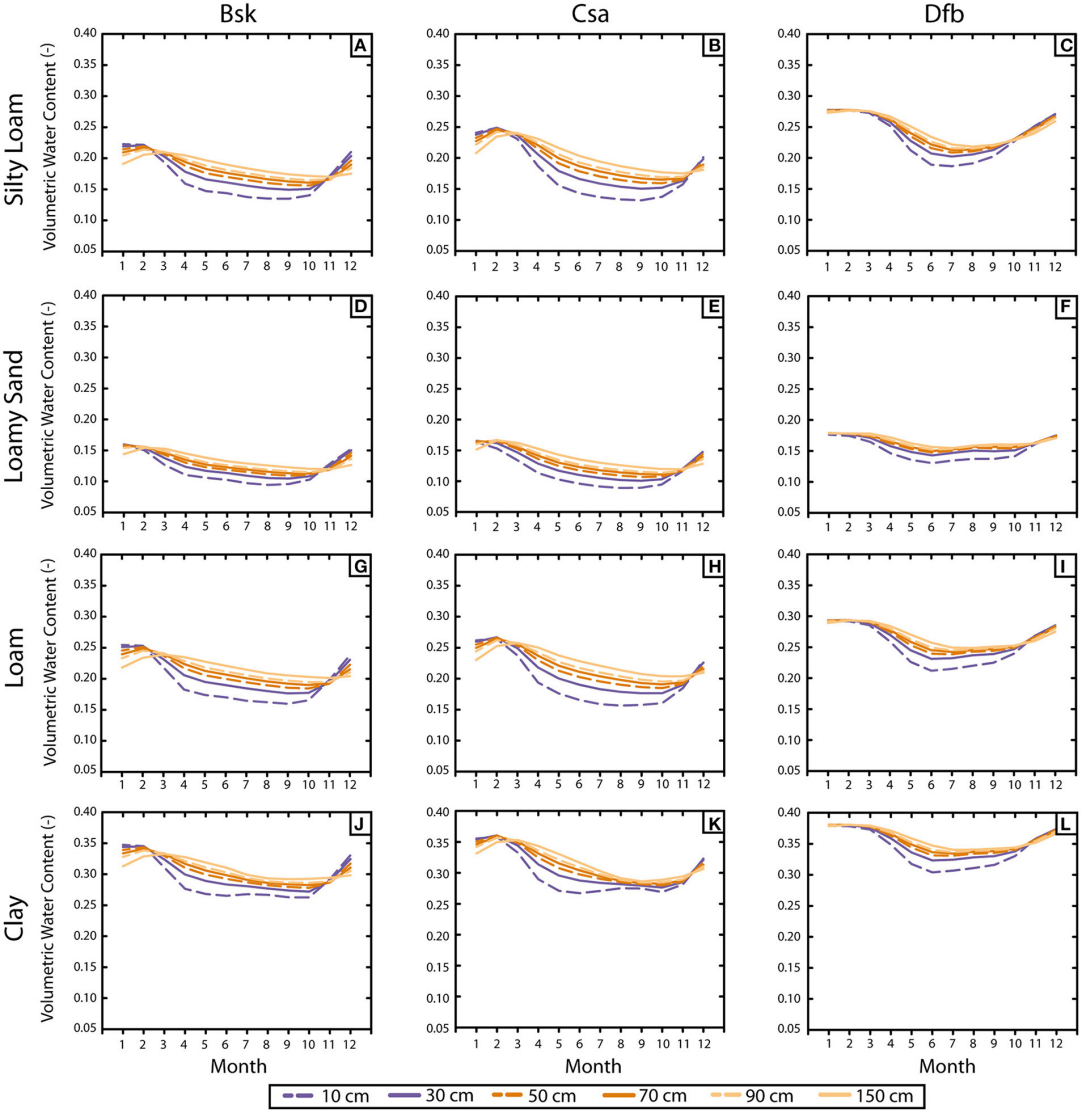
**(A–L)** Monthly average of volumetric water content, VWC, for each combination of soil-climate for the uppermost 1.5 m depth.

As a consequence of these climate features, the monthly distribution of the volumetric water content (VWC) shows a completely different pattern (Figure 2). Each subplot includes the monthly average value of the VWC for depths ranging between 10 and 150 cm. A general view reveals the great difference in volumetric water content derived from the soil type, regardless of the climate, ranging from average values of 0.35 in the case of clay soils to <0.15 for loamy sand soils.

Particularly, for silty loam soils, the maximum values of VWC are 0.22 (Bsk), 0.24 (Csa), and 0.27 (Dfb). These values were found in January for the three climates and are almost constant for every depth range until March when they begin to decrease (Figures 2A–C). Then, the shallower VWC becomes lower than the deeper ones. Dfb displays a sharper drop than the others, decreasing up to the minimum on June-August (Dfb: 0.19; Figure 2C), and on July-October (Bsk and Csa: 0.14; Figures 2A,B). As expected, the maximum variations in VWC were observed in the shallowest level (i.e., 10 cm). Differences between the lowest and deepest depths range from 0.03 (Dfb) to 0.05 (Csa) at the minimum VWC.

The VWC evolution in loamy sand soils is similar to the silty loam soils. Maximum VWC values are reached in January-February. Minimum values are achieved gradually from July-September. The difference between the lower and deeper levels at the minimum is about 0.03–0.04 for Bsk and Csa (Figures 2D,E) and 0.02 for Cfb (Figure 2F).

For loam soils (Figures 2G–I), the VWC evolution in Bsk and Csa's climates features silty loam and loamy sand soils. The decrease is gradual, mainly from April to August. Maximum VWC values (0.25–0.26) are reached on January-February, whereas, the minimum VWC is achieved on July-October with a VWC = 0.16. The difference between the lower and deeper levels at the minimum is about 0.05–0.06, respectively. On the other hand, Dfb's VWC evolution is similar to loamy sand soil, which experiences a more abrupt fall than the other two. The maximum value (0.29) is reached in January-March whereas the minimum VWC is 0.21 in June, with a difference between the shallowest and the deepest levels of about 0.05. After the summer, VWC values in the shallowest depths become higher than the deepest ones and occur in November (Bsk, Csa; Figures 2G,H) and October (Dfb; Figure 2I).

Clay soils behave differently from the other investigated soils. On the one hand, the VWC evolution of Bsk and Csa climates differ in two issues. First, they present two minimums: one in May-June and the other in September-October, both with a VWC = 0.27 (Figures 2J,K). Second, the differences between the deepest and shallowest levels are different for each minimum; thus, in the first one, the VWC difference is about 0.05, whereas the difference of the second one is lower (0.02) for both climates. Maximum VWC values are reached in January-February (0.34, Bsk; 0.36, Csa; Figures 2J,K). On the other hand, Dfb's VWC evolution (Figure 2L) is almost the same as in loam soils. The maximum value is 0.38, reached on January-March. The minimum is 0.30 in June, with a difference between the shallowest and the deepest levels of 0.04. As in the loam soils, after the summer VWC values in the shallowest depths turn higher than the deeper ones and occur in November (Bsk, Csa; Figures 2J,K), and October (Dfb; Figure 2L).

### Gas Permeability

**Figure 3:**
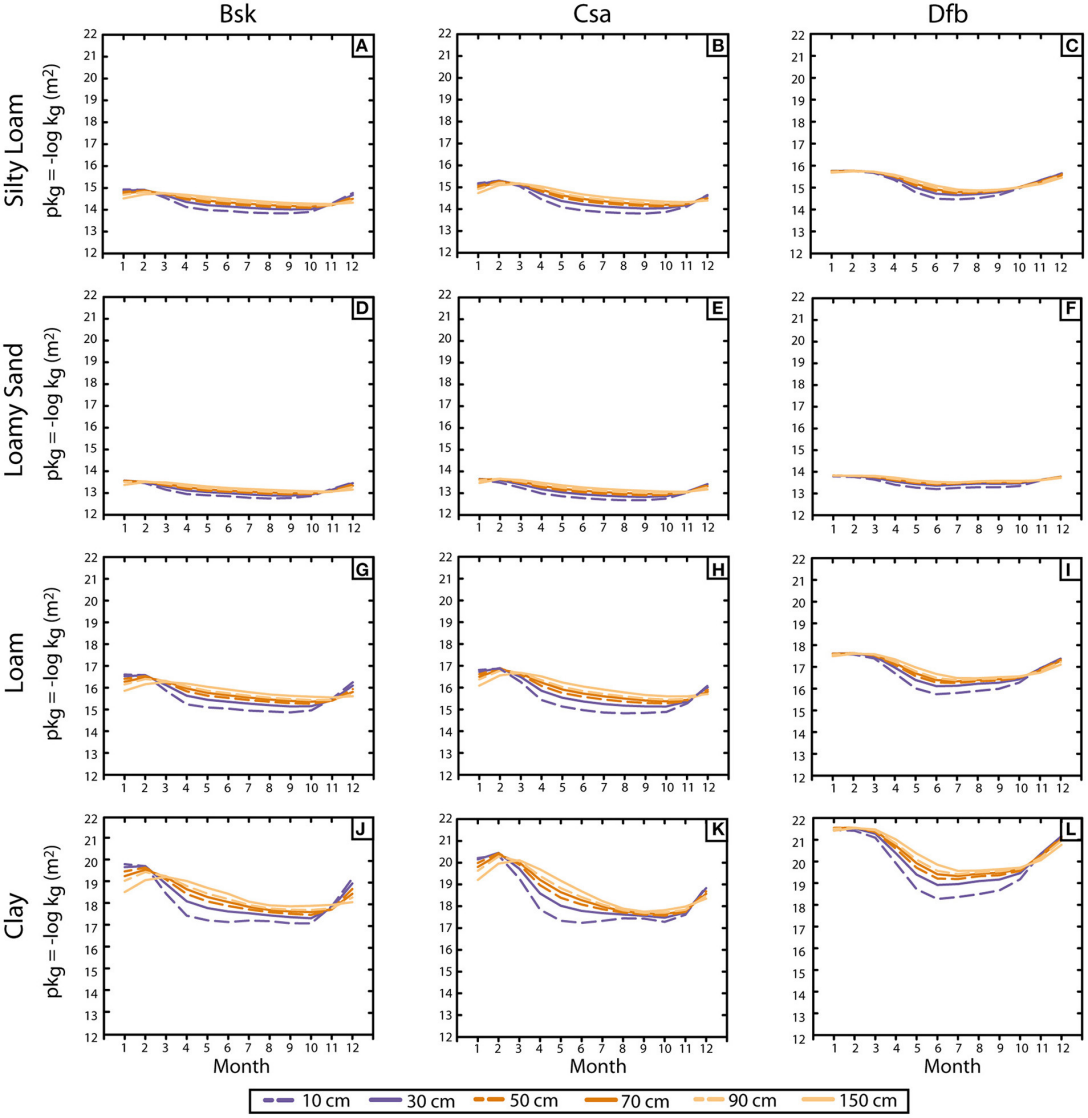
**(A–L)** Monthly average of soil gas permeability (pkg = − log kg) for each combination of soil-climate for the uppermost 1.5 m depth.

Figure 3 displays the monthly average values of gas permeability, expressed as pk_g_, for depths ranging from 10 to 150 cm. High pk_g_ values correspond to lower values of permeability. The pk_g_ evolution shows a flatter annual distribution compared to VWC evolution (Figure 2), except for clay soils, where annual differences between the maximum and the minimum pk_g_ through the year remain very significant.

In the case of the silty loam, the maximum-minimum values of pk_g_ are 14.9–13.8 (Bsk; Figure 3A), 15.3–13.8 (Csa; Figure 3B), and 15.8–14.4 (Dfb; Figure 3C), obtained for the shallowest level. These pk_g_ differences are higher than 1, which means that the effective movement during the month with the highest permeability (August) is more than 10 times the soil gas permeability during the month with the lower values (January). The interannual difference reduces significantly if we focus on the gas permeability at 1 m deep, leading to a difference of pk_g_ 0.6 in the case of Bsk climate and 0.9 for Csa and Dfb climates.

For loamy sands, the maximum and minimum pk_g_ values for the shallowest level are 13.5–12.8 (Bsk; Figure 3D), 13.6–12.7 (Csa; Figure 3E), and 13.8–13.2 Dfb (Figure 3F). This type of soil presents the smallest difference between the maximum and the minimum values observed in pk_g_ along the year as well as the small difference between average values obtained from different climates. The narrow range of the observed VWC in all three climates (Figures 2D–F) causes an even narrower range in pk_g_.

In the case of loam, the maximum and minimum pk_g_ values for the shallowest levels are 16.6–14.9 (Bsk; Figure 2G), 16.8–14.8 (Csa; Figure 2H), and 17.6–15.7 (Dfb; Figure 2I). It implies that in the case of the Csa soil, for example, the soil gas permeability during September is 100 times higher than the gas permeability during the month with the smallest permeability, which is January-February. This fact shows the importance of considering the soil water content when evaluating the risk of radon gas exposure because this risk is, following the same proportionality, 100 times higher in summer than in winter.

Clay is the soil type that presents the highest differences in its extreme values of pk_g_. The maximum pk_g_ values for the shallowest levels are 19.9 (Bsk; Figure 3J), 20.3 (Csa; Figure 3K), and 21.5 (Dfb; Figure 3L), and the minimums are 17.1 (Bsk), 17.2 (Csa), and 18.3 (Dfb). These differences establish that the radon exposure risk considerably increases from summer (the period of the year with the highest gas permeability) and winter (the period with the lowest values of gas permeability). Focusing at one-meter depth, these differences between the periods of the year are reduced, although permeability differences are higher than 100 in the case of Csa and Dfb climates.

## Discussion

Results reveal that climate strongly affects soil gas permeability and they must be considered as an additional factor during the evaluation of radon exposure risk. The impact of climate and texture defines the soil water content dynamic. It may cross the boundary between two radon risk categories, and consequently, may change the radon risk category towards lower risk categories.

Focusing on the specific combinations of soils and climates, we can conclude that the difference in soil gas permeability between the maximum and the minimum value along the year is very much impacted by soil type. As a consequence, the importance of climate in radon risk variation along the year will depend on which type of soil we are dealing with.

Clay soils are the ones that show the higher differences from one climate to another: average pk_g_ differs almost two degrees of magnitude between Dfb and Bsk. As a general rule, coarse soils undergo a smaller impact on the climate. This fact comes from the narrow range of soil water content that shows coarse soils when compared with the bigger range that is more common in fine soils.

This different behaviour of the soil types when comparing different climates can also be extrapolated to the interannual variation within the same climate. Coarse soils show a small difference in volumetric water content and the subsequent gas permeability and, in those cases, the consideration of climate setups does not provide significant improvements when calculating the radon exposure risk. The opposite situation is true when dealing with finer soils, such as clay. In these soils, the interannual variation of pk_g_ and the consideration of climate should be compulsory, as we have illustrated in the studied combinations in which the gas permeability may change between 100 and 1,000 times between different months.

Results conduct several potential applications that may enhance the estimation of the radon potential at different temporal and spatial scales. Considering radon surveys, the measurement and correction of the water content in the gas permeability will lead to more reliable radon movement. Field measurements could be normalized to a reference soil water content or to the site-specific mean soil water content, which includes the expected variations of mean soil water content throughout the study area (10). Moreover, the elaboration of a geogenic radon potential (GRP), both for a specific region and for a GRP largescale mapping, could be used for radon permeability estimations if a representative water content and soil texture data are available.

Our findings also may be considered for the assessment of indoor radon. Recently, several investigations have been attempted to link indoor radon concentrations and geogenic radon information [e.g., (7, 25–29)]. For example, in the indoor radon map of North Ireland (30) authors used geology characteristics, airborne gamma-ray spectrometry data and soil geochemistry. They also highlighted the relevance of how soil gas permeability may elevate radon potential where geological materials do not have high radon concentrations. Later, (31) remarked that soil radon concentration and permeability provided the best indoor radon estimations.

## Conclusion

In the present study, we have studied the impact of three common European climates in combination with four soils ranging from very fine to very coarse when evaluating the soil gas permeability in terms of radon exposure risk. From our results, we can conclude that the impact of climates on gas permeability is different according to the soil analysed. As a general rule, the impact of climate is small, and it may be neglected without falling into big inaccuracies when studying gas permeability in the case of sandy soils. On the other hand, the impact of climate is very important on gas permeability when we are dealing with finer soils. In these soils, the consideration of climate may produce differences in gas permeability between 100 and 1,000 times between different climates or even between different seasons along the year with the same climate. Results conclude that the presence of water content may produce that radon risk crosses the boundary between two different categories, consequently changing towards lower risk.

Findings from the present study should be considered in regulations and normative dealing with the radon risk estimation. Moreover, the measures to reduce the radon risk should not be equal for different places just because the geology or the edaphology are the same. Additionally, results from the present study provide knowledge to define new strategies for the elaboration of more reliable GRP largescale maps considering the climate and soil texture.

## Data Availability Statement

The original contributions presented in the study are included in the article/supplementary material, further inquiries can be directed to the corresponding author/s.

## Author Contributions

SG-O: investigation and writing—original draft. JV-A: methodology, investigation, and writing—original draft. CP: validation, investigation, and writing—review and editing. DB: conceptualization, investigation, writing—review and editing, and supervision. All authors contributed to the article and approved the submitted version.

## Funding

This work was supported by the Spanish Ministry of Science, Innovation, and Universities [grant number RTI2018-099052-BI00] and Regional Governments of Comunidad Valenciana (Spain) [grant number AICO/2020/175]. A pre-doctoral research fellowship (PRE2019-088294) was awarded to SG-O for the project RTI2018-099052-BI00.

## Conflict of Interest

The authors declare that the research was conducted in the absence of any commercial or financial relationships that could be construed as a potential conflict of interest.

## Publisher's Note

All claims expressed in this article are solely those of the authors and do not necessarily represent those of their affiliated organizations, or those of the publisher, the editors and the reviewers. Any product that may be evaluated in this article, or claim that may be made by its manufacturer, is not guaranteed or endorsed by the publisher.

## Abbreviations

Bsk: cold semi-arid climate
Csa: temperate Mediterranean climate
Dfb: humid continental climate
ET_0_: potential evapotranspiration
GRP: geogenic radon potential
PTF: pedotransfer function
USDA: United States Department of Agriculture
VWC: volumetric water content.
